# *In Vitro* HIV-1 LTR Integration into T-Cell Activation Gene CD27 Segment and the Decoy Effect of Modified-Sequence DNA

**DOI:** 10.1371/journal.pone.0049960

**Published:** 2012-11-28

**Authors:** Rei Ohmori, Tatsuaki Tsuruyama

**Affiliations:** Department of Pathology, Center for Anatomy, Pathology, and Forensic Medical Study, Graduate School of Medicine, Kyoto University, Kyoto, Japan; McGill University AIDS Centre, Canada

## Abstract

Integration into the host genome is an essential step in the HIV-1 life cycle. However, the host genome sequence that is favored by HIV-1 during integration has never been documented. Here, we report that CD27, a T cell activation gene, includes a sequence that is a target for *in vitro* HIV-1 cDNA integration. This sequence has a high affinity for integrase, and the target nucleotides responsible for this higher affinity were identified using a crystal microbalance assay. In experiments involving a segment of the CD27 gene, integration converged in the target nucleotides *and* flanking sequence DNA, indicating that integration is probably dependent upon the secondary structure of the substrate DNA. Notably, decoy modified CD27 sequence DNAs in which the target nucleotides were replaced suppressed integration when accompanying the original CD27 sequence DNA. Our identified CD27 sequence DNA is useful for investigating the biochemistry of integrase and for *in vitro* assessment of integrase-binding inhibitors.

## Introduction

After infection by the human immunodeficiency virus (HIV)-l retrovirus, DNA synthesis begins in the cytoplasm of the infected cell and may be completed before or after entry into the nucleus. Integrase, which is encoded by the retroviral genome, cleaves the viral DNA termini in preparation for attachment of the proviral DNA to the host DNA. In the cytoplasm, the viral DNA forms a nucleoprotein complex and enters the nucleus. The site of retrovirus integration into the host DNA has long been believed to be random.

The vast majority of retroviral integration studies published to date have involved infection of cultured cells with retroviruses followed by identification of the sites of integration into the host cell genome. Retroviral integration into the host genome is not an entirely random process, and the integration site preference varies among retroviruses. There are reports that active genes are the preferential targets of HIV integration [1]. A recent study reported that integration in resting CD4+ T cells occurs more often in regions that may be suboptimal for proviral gene expression [2]. Studies of HTLV-1 integration sites in human HeLa cells have shown that HTLV-1 does not specifically target transcription units or transcription start sites [3]. On the other hand, weak palindromic sequences are a common feature of the sites targeted by retroviruses [4]. The tendency of integrase to form dimers or tetramers is consistent with a preference for integration at palindromic sequences. Although available evidence suggests that integration of retroviruses into the host genome is a non-random process, the actual target DNA sequence has not been reported [5], [6]. Here, we report the sequence of the DNA segment within the coding region of the human CD27 gene as determined through *in vitro* analysis of HIV-1 integration [7].

A thorough analysis of previously reported HIV-1 T cell genome integration sites found in Genbank revealed that in human genes most integration sites are located in noncoding regions and that integration into coding regions is rare. We found that CD27 is one of the few genes in which HIV-1 integrates into the coding region. The CD27 gene is involved in T cell activation; therefore, integration into this gene may influence differentiation of host T cells by altering expression of this gene. Schroeder *et al.* reported that HIV-1 prefers to integrate into transcriptional activation genes [1]. We hypothesized that HIV-1 integration into the T cell genome occurs during transcriptional activation of the CD27 gene. By characterizing the features of the integration sequence, we may enhance understanding of the integration process. Because we found that the CD27 sequence segment into which HIV-1 integrates includes a palindromic feature, which was anticipated in a previous report, we utilized a biophysical technique, the quartz crystal microbalance (QCM) assay, to examine the affinity of integrase for this sequence segment. Integration into the CD27 segment target sequence was assessed using an *in vitro* integration assay.

## Materials and Methods

### Identification of the Integration Site in CD27 Locus

HIV-1 integration sites were meta-analyzed using previously reported sites in the DNA of HIV-1–infected cells. Analysis of *Homo sapiens* PAC clone RP11-102E24 revealed that HIV-1 integrates into the CD27 locus in the genome of HIV-1–infected clone SupS1 human lymphoma T cells [Genbank Accession No. AF038363] [6], [8]. The integration site was identified in the first intron located between the first and second exons of CD27.

### *In vitro* Integration

*In vitro* integration was assessed essentially according to a previously reported protocol [9]. Briefly, 150 ng of U3′-R-U5′-U3′-R-U5′ tandem LTR HIV-1 cDNA was added to 50 ng of recombinant HIV-1 integrase in 10 µL of binding buffer and incubated for 1 h at 30°C. The binding buffer consisted of 1-0.1 mM MgCl_2_, 80 mM potassium glutamate, 10 mM mercaptoethanol, 10% DMSO, and 35 mM MOPS (pH 7.2). The target human CD27 DNA was ligated into circular pCR2.1 TOPO vector plasmid DNA (Invitrogen, Carlsbad, CA), and 500 ng of the ligation product DNA was used as the target DNA for the assay. Truncated target DNA or target DNA with deleted nucleotides was also ligated into circular pCR2.1 TOPO vector plasmid DNA. HIV-1 integrase was kindly provided by Dr. Tomokazu Yoshinaga [10]. The pCR2.1 plasmid containing integrated HIV-1 proviral DNA was used to transform *E. coli* cells, from which the plasmid DNA was subsequently extracted and sequenced.

The sequence of the target DNA was modified in several ways for the *in vitro* integration assay. Nucleotides within the target segment and nucleotides in the presumed DNA stem were replaced. The details of target sequence and the replacement nucleotides were represented in text. The percentage of integration was estimated by determining the copy number of the products of integration into the target DNA compared to the copy number of the products of integration into the whole-sequence DNA (i.e., the target DNA plus the plasmid), according to previously reported methods [7], [9], [11]. First, the number of *E. coli* colonies exhibiting integration into the target segment was determined. Second, the number of *E. coli* colonies exhibiting integration into the whole-sequence DNA was determined. The ratio of the former count to the latter count was considered the percentage of integration into the target segment. For enhancement of integration efficiency, a 378-bp target DNA consisting of 6 repeats of the 63-bp CD27 sequence was utilized following ligation to the pCR2.1 TOPO vector plasmid DNA that was 3.9-kb pairs long. One repeat CD27 sequence was not sufficient for in the present *in vitro* integration study, probably because the target segment was too short to be recognized by integrase.

### Quartz Crystal Microbalance (QCM) Assay

A quartz crystal microbalance assay was used to evaluate the affinity of integrase for the target DNA. Changes in the frequency were monitored following the binding of 50 ng of integrase to 200 ng of target DNA in a terminal crystal oscillator. The oscillator consisted of an electrode equipped at both sides with an AT-angle cut crystal. Upon application of an alternating electric field, oscillation of the target DNA-binding electrode in the direction of horizontal shear occurs on the crystal surface (thickness-shear-mode resonator). The relationship between the mass of the DNA deposited on the electrode and the frequency of oscillation is represented by the Sauerbrey equation (*), which shows that the frequency decreases as the mass of material deposited increases:
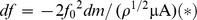
The left side of the equation (*df*) represents the change in the frequency, where *f_0_* represents the resonant frequency; *ρ* represents the density of the crystal; represents the shear stress; *dm* represents the change in the mass associated with integrase binding; and *A* represents the active area of the electrode. Therefore, the amount of integrase bound to the target DNA segment could be compared to that bound to the modified target DNA by determining the change in oscillation frequency.

### Thermodynamic Analysis

DNA folding was predicted using the *m-fold* web program (http://mfold.bioinfo.rpi.edu/cgi-bin/dna-form1.cgi) [12]. The biochemical parameter values used in the calculations will be described later.

### Statistical Analysis

The statistical significance of differences was ascertained through the use of unpaired *t*-tests with SPSS statistical software (SPSS, Chicago, IL, USA). A *P* value < 0.01 was considered to be statistically significant.

## Results

### Evaluation of *in vitro* Integration Efficiency and Selectivity for Focal Dinucleotides

**Figure 1:**
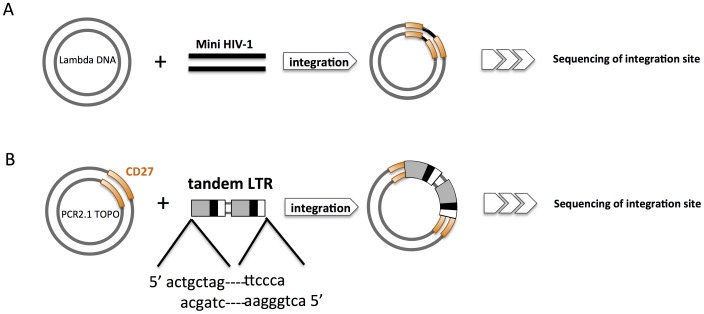
Schematic illustrations of reported protocols for assessing *in vitro* integration. (A) “Mini-HIV-1” consisting of both the 5′- and 3′- ends of HIV-1 proviral DNA is incubated with substrate lambda phage DNA (Bushman). (B) The *in vitro* integration assay described in the present our report, which uses the tandem LTR (U3-R-U5-U3-R-U5) cDNA sequence of HIV-1. The tandem LTR is incubated with a substrate DNA consisting of 6 repeats of the CD27 gene target sequence identified as the *in vivo* HIV-1 integration site.

Previous report frequently utilized artificial DNA sequences or *E. coli*-derived and non-human artificial sequence [9]. A thorough analysis of HIV-1 integration sites accessed from Genbank was performed to identify the coding region of the CD27 gene sequence. Most of the HIV-1 integration sites described in Genbank are located in noncoding regions. There are few integration sites within the coding regions of genes. We used a previously reported assay protocol for the *in vitro* evaluation of integration selectivity as the basis for our analysis (Fig. 1A) [7], [9]. In the current study, we modified the protocol further by omitting the PCR reaction and using the actual human CD27 gene as the target DNA sequence (Fig. 1B) [7], [11].

A 378-bp target DNA consisting of 6 repeats of the 63-bp CD27 sequence 5′-CTGCCCTCTTCAAGGTGGTGCCTCCCCACAGC**T:** ***GCA*** *GG*ATTTCCCTCCTTGCTTGAGCTCAG-3′ was utilized as the target sequence. The integration site is denoted by “:” in the above sequence, and the 5 bases duplicated by HIV-1 integration are shown in italics. The underlined bases CTGCCCTC vs. GAGCTCAG and CAAGGTGGTGC vs. ***GCA*** &CCTCCTTG were palindromic with each other.

**Figure 2:**
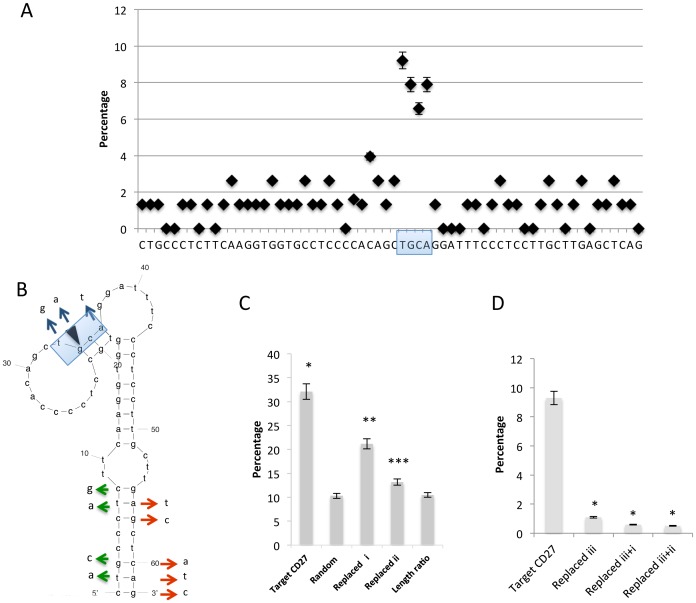
*In vitro* integration site in the target CD27 sequence DNA. (Α) Percentage of integration at individual sites in the target CD27 sequence DNA. A shaded box indicates the frequent integration site in the in vitro integration assay in (B). (B) Anticipated secondary structure formation in the target CD27 sequence DNA as determined by *m-fold* analysis. An arrowhead indicates the integration site in the previously reported target nucleotide (Genbank Accession No. AF038363) [6]. Blue, green, and red arrows represent mutations of replaced *i,* replaced *ii*, and replaced *iii*, respectively. Gibbs’ free energy is given by *ΔG = ΔH -TΔS*, where *ΔG* = −7.34 kcal/mol at 37°C, *ΔH* = −114.80 kcal/mol, *ΔS* = −346.4 cal/(K·mol), T_m_ = 58.1°C under ionic conditions in which [Mg^2+^] = 1.0 mol/L. The same concentration of Mg^2+^ was used in the *in vitro* integration assay. A shaded box indicates the frequent integration site in the *in vitro* integration assay. (X) Percentage of integration at various sites in the target CD27 sequence DNA, control random DNA, and modified DNAs (replaced *i* and replaced *ii*). Length ratio percentage indicates the ratio of percentage integration into the target sequence DNA to that of integration into the whole substrate DNA. The percentage integration into the CD27 sequence DNA was significantly higher than that into random sequence and replaced DNAs (*i*) and (*ii*) (*, **, ***, *P* < 0.01). (Δ) Percentage of integration into the target sequence DNA relative to the percentage of integration into the whole substrate DNA (*P < 0.01 vs. target CD27 DNA segment). The site of the nucleotide replacement in the Replaced (*iii*) segment is shown by the red arrow in Fig. 2A. Letters next to the arrows in 2A denote the replacement nucleotides. Results are representative of 5 independent assays. The means ± SD are shown. The notations (*iii*)+(*i*) and (*iii*)+ (*ii*) signify segments with both types of modification.

Integration sites in *in vitro* integration assay were localized within the **T: GCA** site (Fig. 2A, indicated by a shaded box). As a result, the ratio of the percentage of integration into the target sequence to that of integration into the whole substrate DNA (plasmid plus target sequence) was approximately 3.2 times greater than the percentage obtained in an assay using random sequences (10.5%; *P* < 0.01 vs. random), which was nearly equivalent to the ratio of the percentage of integration into the target 360-bp DNA to that of integration into the whole 3.9-kb DNA.

Subsequently, an *in vitro* integration assay using the target CD27 gene in which the **T: GCA** sequence was replaced with GACT was then performed (Fig. 2B and C, “replaced *i*”). The percent integration into the replaced *i*-target segment was significantly lower than the percent integration into the native CD27 sequence DNA (**P < 0.01, vs. native; Fig. 2C). The sequence 5′-CACCCCAGTTCAAGGTGGTGCCTCCCCACAGC**T:** ***GCA*** *GG*ATTTCCCTCCTTGCTTGAGCTCAG-3′ was also evaluated using this assay. The underlined bases were replaced in order to disrupt the palindrome (Fig. 2B and C, “replaced *ii*”). Disruption resulted in a significant reduction in the integration percentage relative to the native sequence (***P < 0.01 vs. native; Fig. 2C). Another replaced sequence, 5′-CTGCCCTCTTCAAGGTGGTGCCTCCCCACAGC**T:** ***GCA*** *GG*ATTTCCCTCCTTGCTTGTCCTATC-3′, within the target sequence (see Fig. 2B) (replaced “*iii*”) also resulted in a significant reduction in the integration percentage, due to disruption of the palindrome sequence motif (*P < 0.01 vs. native; Fig. 2D).

### Affinity of Viral Integrase for Target Sequences

**Figure 3:**
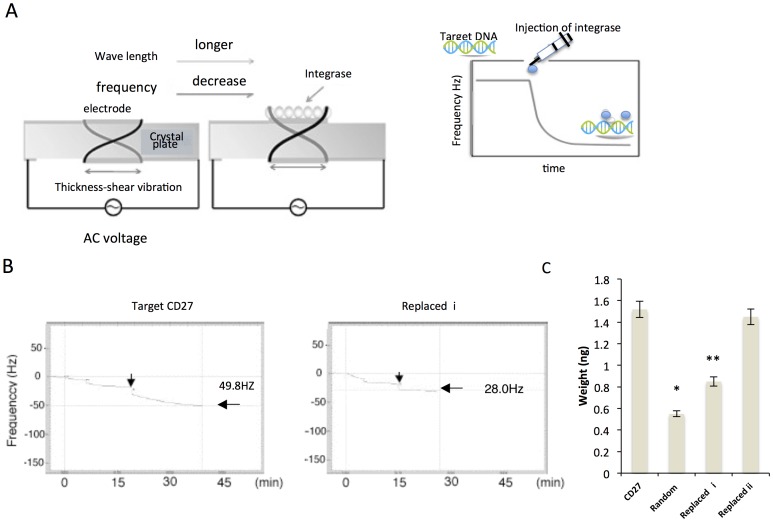
Assessment of integrase binding using a quartz crystal microbalance. (A) Scheme depicting the quartz crystal microbalance assay. DNA is deposited on the electrode. (B) Representative graphs of results of assays using target CD27 DNA and replaced *i* DNA. Downward arrows represent the frequency (Hz) at the plateau phase after integrase binding. (C) Graph showing the weight of integrase bound to CD27 target, random, replaced *i*, and replaced *ii* DNAs which were fixed onto the QCM sensor chip. (*, **P < 0.01). Results are representative of 5 independent assays. The means ± SD are shown.

Quartz crystal microbalance (QCM) technology was applied to measure the affinity of viral integrase for host CD27 DNA. Integrase binding activity was evaluated by determining the weight (ng) of integrase bound to the oscillator-detection sensor (Fig. 3A). In addition, the 5′-**T**:**GCA**-3′ sequences in the repeat unit segments were removed and replaced (“replaced *i* and *ii*) and the resulting products were examined using the assay. The weight of integrase that bound to the replaced *i* modified DNA was lower than the weight of integrase that bound to the native DNA sequence, but the difference was not significant, strongly suggesting that the binding affinity of integrase is dependent upon the 5′-**T**:**GCA**-3′ sequence in the target DNA (Fig. 3B).

### Suppression of Retroviral Integration by Modified Target Sequence DNAs

**Figure 4:**
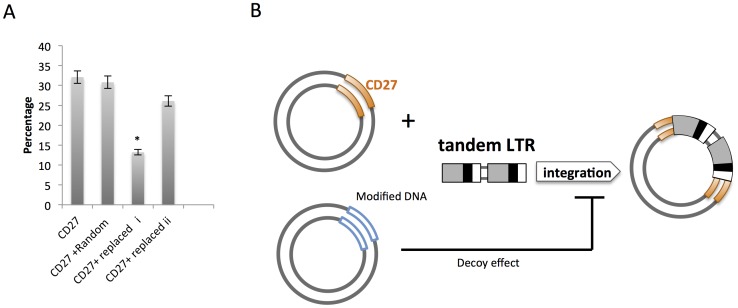
Decoy effect of the CD27 modified sequence. (A) The percentage of integration into the native target DNA was significantly suppressed in the presence of the modified DNAs replaced *i* and replaced *ii* (*, **P < 0.01). Results are representative of 5 independent assays. The means ± SD are shown. (B) Scheme depicting the proposed mechanism of the decoy effect of the modified DNA.

Modified substrate DNA (replaced *i* or replaced *ii*) was mixed with an equal concentration of native target CD27 DNA and an *in vitro* integration assay was performed. Unexpectedly, integration into the native target DNA was significantly repressed in the presence of modified replaced *i* DNA (Fig. 4A, *P < 0.01). The modified sequence DNA interfered with viral integration (Fig. 4B), which is consistent with our previous report [7].

## Discussion

The biochemical properties of interaction between HIV-1 integrase and its target DNA were investigated through the development of a new *in vitro* integration assay using target gene DNAs. We found that the target nucleotide sequence TGCA located at the integration site appears to influence the integration process. We also replaced nucleotides in the presumed stem structure in the CD27 segment to examine whether inhibition of secondary structure formation had an effect on integration. The fact that replacing nucleotides in the presumed stem structure resulted in a significant suppression of integration into in the target segments suggests that the secondary structure formed by the native CD27 sequence DNA renders the target segment more accessible for integrase. By application of fluorescence resonance energy transfer (FRET) Katz et al. previously noted the possibility that the secondary structure may be formed by the target double-stranded DNA during integration [13], [14]. Indeed, figure 2B shows the secondary structure anticipated by *m-fold* analysis of above shown sequence [12], which revealed that the integration site (indicated by a shaded box) was located at the corner of a loop attached to a long stem structure. The replacement of bases was anticipated to disrupt the stem of the presumed structure.

In addition, the fact that HIV preferably integrates into transcriptionally active genes [1] suggests that the target segment used in the present study, which is part of a gene involved in T cell development, is probably transcriptionally active. As such, the segment may therefore be accessible to DNA-binding proteins such as transcription factors or components of the transcriptional apparatus. It is also possible that the double strand in the target segment may be rewound into a single strand following formation of the loop-like structure by hybridization within the strand. Therefore, it appears that both the focal nucleotides at the integration site and the secondary structure generated by the sequences flanking the integration site play a role in determining the accessibility for integrase.

The QCM assay was used to directly determine which segment of the target DNA sequence is preferentially bound by integrase [15]. The results of QCM assays demonstrated that approximately twice as much integrase was bound to CD27 DNA, including the TGCA sequence, than was bound to the modified DNAs we examined. Studies indicate that HIV-1 proviruses and other proviruses such as HTLV-I and MLV share the dinucleotide motif 5′-CA and 5′-TG at their termini [16]. It is therefore likely that interaction between the TGCA sequence in the target DNA and the viral DNAs is a common occurrence.

In our previous study [7], we found that the modified target sequence favored in HIV-1 cDNA integration affected integration into the native target sequence. The cause of this *in vitro* interference, termed the *decoy effect*, remains unclear. One plausible explanation is that the modified DNA segment has some affinity for the HIV-1 integrase-cDNA complex, and that this low affinity interferes with integration through competition. This possibility is supported by QCM assay results indicating that the affinity of the modified segments for the integrase complex is about half of that of the native DNA segment. As a result, the modified sequence DNA competes with the native DNA for integrase.

The CD27 antigen is involved in the activation of T cells and plays a role in the infection of T cells by HIV-1. Integration of HIV-1 into CD27 disrupts the CD27 translational region. Integration into the genome of CD4+ T cells renders the host cell unable to differentiate through the CD27 signal [17]. CD27 plays a supportive role in T(H)1 differentiation *in vivo*, without modulating the classical T(H)2 response. In addition, CD27 instructs CD4+ T cells to provide help for the memory CD8+ T cell response after protein immunization [18]. These observations suggest that when HIV-1 integrates into CD27, the CD4+ T cell help response is blunted, resulting in insufficient CD8+ cytotoxic T cell-mediated removal of HIV-1–infected CD4+ T cells. Altered T cell differentiation was shown to be associated with the loss of CD27 in HIV-infected Indians [19]. HIV-1–specific memory CD4+ T cells are phenotypically less mature. After highly active anti-retroviral therapy (HAART), HIV-1–specific CD4+ T cells are enriched for CD27+ CD28 (−)-expressing cells, a rare phenotype, reflecting an early intermediate stage of differentiation [20]. If integration into CD27 affects the differentiation or activation of host CD4+ T cells, such arrest may contribute to the observed lack of help by CD4+ T cells and the development of AIDS.

Further detailed studies are needed to more completely elucidate the precise biochemical properties of *in vitro* retroviral integration. It is possible that such studies may reveal the existence of a more favored target segment. The findings arising from the current study may help facilitate a greater understanding of retroviral integration.
